# Equine Gram-Negative Oral Microbiota: An Antimicrobial Resistances Watcher?

**DOI:** 10.3390/antibiotics12040792

**Published:** 2023-04-21

**Authors:** José Pimenta, Ana Rita Pinto, Maria José Saavedra, Mário Cotovio

**Affiliations:** 1 Department of Veterinary Sciences, Antimicrobials, Biocides & Biofilms Unit (A2BUnit), University of Trás-os-Montes and Alto Douro, 5000-801 Vila Real, Portugalmcotovio@utad.pt (M.C.); 2 CECAV-Veterinary and Animal Research Center and Associate Laboratory for Animal and Veterinary Sciences (AL4AnimalS), University of Trás-os-Montes and Alto Douro, 5000-801 Vila Real, Portugal; 3 CITAB-Centre for the Research and Technology of Agro-Environmental and Biological Sciences and Institute for Innovation, Capacity Building and Sustainability of Agri-Food Production (Inov4Agro), University of Trás-os-Montes and Alto Douro, 5000-801 Vila Real, Portugal

**Keywords:** equine, oral microbiota, multidrug resistance, antimicrobials, One Health

## Abstract

Horses are considered as reservoirs of multidrug resistant bacteria that can be spread through the environment and possibly to humans. The aim of this study was to characterize the oral Gram-negative microbiota of healthy horses and evaluate their antimicrobial susceptibility profile in a One Health approach. For this purpose, samples were collected from the gingival margin of healthy horses, free of antimicrobial therapy, cultured in selective mediums, identified, and tested for antimicrobial susceptibility. Fifty-five Gram-negative isolates were identified, with 89.5% being zoonotic and 62% affecting humans, which were also found commonly in the environment. Forty-eight isolates (96%) were MDR. The phenotypic resistance presented as higher to macrolides (81.8%), β-lactams (55.4%), and quinolones (50%), and lower to sulfonamides (27.3%), tetracyclines, and amphenicols (both with 30.9%). In total, 51.5% of the isolates presented resistance to carbapenems. In addition to being the first report on the commensal oral microbiota of horses and respective susceptibility profile, this study highlights the horse as a valuable sentinel that can control the evolution and transmission of multidrug-resistant bacteria between the “One Health triad” since it is in contact with humans, other animals, and the environment, in different geographic locations.

## 1. Introduction

The microbiome of the horse is an increasingly explored field of research. However much of this research focuses on the microbiome of the gastrointestinal tract and its role in the metabolism of the horse at various levels [1]. The literature regarding oral microbiota in recent decades is scarce. However, since certain oral pathologies are related to infections, knowledge of the commensal microbiota may help us to understand the development and changes that occur during these diseases [2]. In addition to the health of the horse itself, knowledge of the oral microbiota is of interest from a public health point of view. Saliva is a medium for the dissemination of various agents and the multidrug resistances they entail. Humans are in very close contact with the mouth of horses, either from the veterinary perspective during dentistry procedures or from the equestrian perspective where there is a great deal of handling of the horses’ mouths. Because of this, it is important to note that, due to close contact with horses, about 100,000 emergencies that occur in the United States are due to horse accidents and, of these, 3–4.5% are due to horse bites [3]. As stated by Langley et al. (2009), these bites can transmit zoonotic agents and although the wounds initially appear harmless, a large proportion progress to states of infection with severe complications. For this reason, the United States considers animal bites a public health problem. Due to the existing complications, besides the study of the microbiome, it is also important to promote studies on the antimicrobial susceptibility profile of these strains regarding human and veterinary commonly used antibiotics [3].

According to the World Health Organization (WHO), antimicrobial resistance has become one of the most pressing health issues of our time, due to increasing resistance to commonly and uncommonly used antimicrobials, which can lead to life-threatening consequences [4,5]. The problem is further exacerbated by the lack of newly researched and approved antibiotic classes for veterinary medicine and precisely for horses. To solve this issue, a more comprehensive strategy must be used that considers the importance of not only the bacteria that directly affect humans, but also animal pathogens, the commensal microbiota of humans and animals, the zoonotic agents, and the environmental strains that easily spread among different ecosystem compartments that create niches of antimicrobial resistance. Several countries around the world have tackled this problem by applying measures and laws that cover a wide range of stakeholders—pet owners, veterinarians, human health professionals, farmers, and all ordinary citizens. This integrated tactic, where no one is left out, is called the “*One Health*” approach. By definition, One Health is “the collaborative effort of multiple health science professions, together with their related disciplines and institutions—working locally, nationally, and globally—to attain optimal health for people, domestic animals, wildlife, plants, and our environment” [6]. The results show that it appears to be successful when it comes to reducing antibiotic resistance rates [7,8]. However, there is still a long way to go. Equine practitioners in particular face some difficulties and challenges when it comes to using antibiotics wisely, such as accurate and prompt pathogen detection, because they frequently work in the field rather than in hospital facilities, the small number of antimicrobial drugs available specifically for horses, and, regrettably, the incorrect and indiscriminate administration of antibiotics by some owners [9].

The horse has always played a very important role in the history of society, whether as a working animal, a sports animal, or just a companion. In addition, it plays a fundamental therapeutic role in animal-assisted therapy. Over time, the intimate contact between humans and domestic horses has increased exponentially. Regarding these intimate connections between humans and horses, it is essential to properly diagnose infectious diseases and antimicrobial resistance that may impact both people and horses. This is especially true for infections that are highly transmissible, since horses have been linked to several significant zoonotic pathogens that are antibiotic-resistant [10]. In the classification used by the WHO, when addressing antimicrobial resistance, horses are considered companion animals, which enhances the intimate contact with humans [11]. Despite their extreme relevance to the study and in combatting antimicrobial resistance, the importance of this species has been undervalued when compared to other animals [12]. In addition to the classification imposed by the WHO, in several countries, the horse is still a working animal, a production animal, and a consumption animal [13,14]. The close relationship and intimate contact between horses, other animals, humans, and the environment cannot be neglected, since it increases the risk of spreading multidrug-resistant (MDR) bacteria [10]. To make matters worse, several studies in Europe show that healthy horses are carriers and reservoirs of MDR bacteria, with a relatively high prevalence (39–44%), some of them with zoonotic potential [15,16,17]. Since the great majority of antimicrobial classes are used in both humans and animals, and that approximately 75% of human infectious diseases are considered zoonotic, the consequences of a cross-infection with a resistant agent could be tremendous [18,19].

To control the consequences of antimicrobial resistance, actions that include antimicrobial use regulation, infection control, research into alternatives to antimicrobials, and most of all, surveillance, are necessary. The surveillance is a continuous process that needs to be performed in every sector belonging to the One Health triad in order to evaluate the evolution and movement of resistances. Because of all mentioned facts, more studies on the equine microbiota are needed to follow the evolution of antimicrobial resistance, to contribute to the update of control measures, and to optimize the clinical use of these drugs [9,20].

The aim of this study was to characterize the commensal oral Gram-negative microbiota of healthy horses and evaluate their antimicrobial susceptibility profile, in a One Health approach. According to the authors knowledge, there is no similar study in the literature on commensal oral microbiota and the corresponding resistance profile of healthy horses.

## 2. Results

### 2.1. Clinical Information and Bacterial Species Isolated

Thirty healthy horses (14 females and 16 males) between 3 and 23 years old (medium age 12 years old) and several breeds (17 Lusitanos, 5 Warmbloods, 5 Crossbreed, 1 Arabian, 1 Friesian, and 1 Sorraia) were included in this study. Horses were from six different and distant regions of Portugal (approximately 100 km of distance between stables), all from equestrian centers, having contact with other horses, were stabled but had access to the outdoors, and were fed with concentrate and hay three times per day. Most of the horses were sport horses and, therefore, were regularly transported to other equestrian centers throughout Portugal as well as to other countries; others were used in assisted-therapy.

After the isolation process, 55 Gram-negative isolates were identified, including *Escherichia coli* (n = 15), *Pseudomonas fluorescens* (n = 7), *Enterobacter cloacae* complex (n = 5), *Pantoea agglomerans* (n = 5), *Serratia rubidaea* (n = 4), *Aeromonas salmonicida* (n = 3), *Klebsiella pneumoniae* (n = 3), *Pantoea* spp. (n = 2), *Pseudomonas putida* (n = 2), *Burkholderia gladioli* (n = 1) *Enterobacter aerogenes* (n = 1), *Kluyvera intermedia* (n = 1), *Pasteurella pneumotropica* (n = 1), *Pasteurella testudinis* (n = 1), *Pseudomonas paucimobilis* (n = 1), *Serratia plymuthica* (n = 1), *Shigella sonnei* (n = 1), and *Sphingomonas paucimobilis* (n = 1).

### 2.2. Antibiotic Resistance Profile

**Figure 1:**
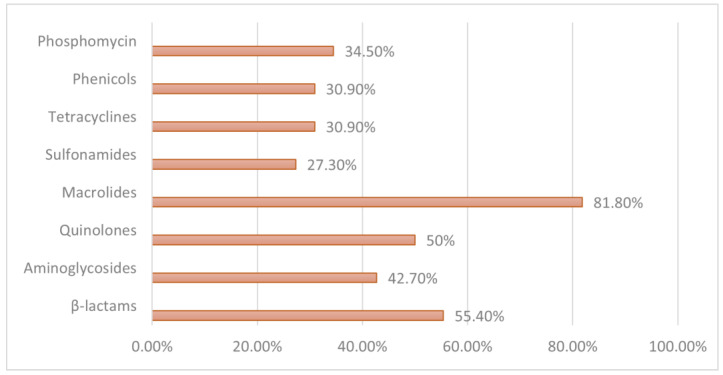
Percentage resistance towards each antimicrobial class.

Forty-eight of the isolates (96%) were MDR. The MDR pattern was distributed by *Escherichia coli* (13/15), *Pseudomonas fluorescens* (7/7), *Enterobacter cloacae* complex (4/5), *Pantoea agglomerans* (5/5), *Serratia rubidaea* (4/4), *Aeromonas salmonicida* (3/3), *Klebsiella pneumoniae* (3/3), *Pantoea* spp. (2/2), *Pseudomonas putida* (2/2), *Burkholderia gladioli* (1/1), *Pasteurella pneumotropica* (1/1), *Pseudomonas paucimobilis* (1/1), *Serratia plymuthica* (1/1), and *Shigella sonnei* (1/1). The phenotypic resistance presented as higher to macrolides (81.8%), β-lactams (55.4%), and quinolones (50%), and lower to sulfonamides (27.3%), tetracyclines, and phenicols (both with 30.9%). It is important to highlight the high percentage of resistance to carbapenems (51.5%). Figure 1 shows the percentage resistance to each antimicrobial class tested.

**Figure 2:**
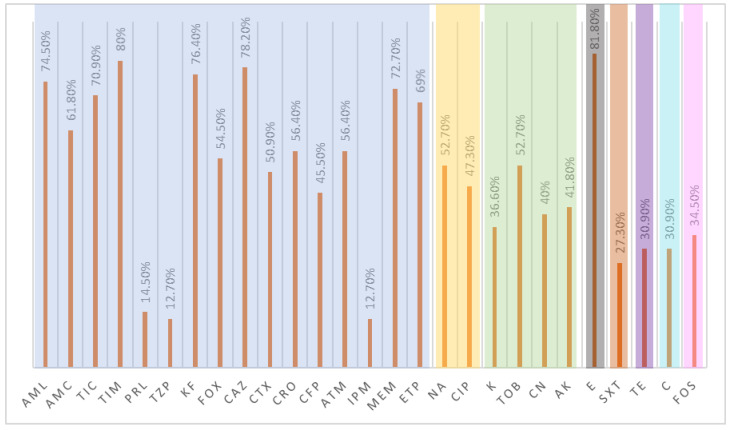
Percentage resistance towards each antimicrobial. β-lactam antibiotics tested included penicillins (aminopenicillins, carboxypenicillins, and ureidopenicillins), cephalosporins (1st and 3rd generations), monobactams, and carbapenems, namely amoxicillin (AML_10_), amoxicillin/clavulanic acid (AMC_30_); ticarcillin (TIC_75_), ticarcillin/clavulanic acid (TIM_85_); piperacillin (PRL_100_), piperacillin/tazobactam (TZP_110_); cephalothin (KF_30_); cefoxitin (FOX_30_); ceftazidime (CAZ_30_); cefotaxime (CTX_30_); ceftriaxone (CRO_30_); cefoperazone (CFP_30_); aztreonam (ATM_30_); imipenem (IMP_10_), meropenem (MEM_10_), and ertapenem (ETP_10_)—dark blue box. Quinolones (nalidixic acid—(NA_30_)/quinolone, ciprofloxacin—(CIP_5_)/fluroquinolone)—yellow box. Aminoglycosides (kanamycin (K_30_); tobramycin (TOB_10_); gentamicin (CN_10_); amikacin (AK_30_))—green box. Macrolides (erythromycin (E_15_) —grey box. Combination of sulfamethoxazole/trimethoprim (SXT_25_)—orange box. Tetracycline (TE_30_)—purple box. Amphenicols (chloramphenicol (C_30_)—light blue box. Fosfomycin (FOS_50_)—pink box.

Regarding individual resistance to each antimicrobial, the overall results were elevated, with erythromycin (81.8%), ticarcillin-clavulanic acid (80%), and ceftazidime (78.2%) presenting the highest percentages of resistance, while piperacillin-tazobactam and imipenem had the lowest percentage, both with 12.7%. Once more, meropenem and ertapenem, two of the carbapenems tested, presented remarkable percentages of resistance (72.7% and 69%, respectively). Figure 2 contemplates the percentage resistance to each antimicrobial tested.

Table 1 presents the resistance of each bacterium with at least two isolates towards the antimicrobial classes tested. Once again, the highest resistance is evident for macrolids, quinolones, β-lactams, and aminoglycosides. Sulfonamides and phenicols show a lower overall percentage resistance in most isolates. *Aeromonas salmonicida* and *Klebsiella pneumoniae* seems to be some of the strains with a higher overall percentage resistance to the antimicrobial classes tested.

Table 2 presents the resistance of the bacteria with only one isolate towards the antimicrobial classes tested. β-lactams presented a more consistent percentage of resistance across all strains. *Burkholderia gladioli* was the bacteria with the highest overall percentage resistance compared to the other bacteria with only one isolate.

## 3. Discussion

There are several studies describing equine microbiota, specially from the gut, but also from other components, such as the ocular conjunctiva and the skin [21,22,23]. However, studies about equine oral microbiota are scarce and the few that exist focus on pathological conditions such as periodontal disease, diastemata, and caries [1,2,24,25]. None of the studies evaluated the commensals nor the antimicrobial resistance profile. This topic of research is clinically relevant since commensal bacteria can act as opportunistic pathogens under pathological situations when defensive mechanisms are compromised [23]. Depending on the resistance profile, bacteria once thought of as commensals might produce infections that are challenging to treat. In general, the microbiota can change over time and between individuals according to geographical location, time of the year, environment, nutrition, contact with other animals, and several other factors [7,26]. Our study evaluated the equine oral Gram-negative commensal microbiota and respective antimicrobial susceptibility profile on several horses that belonged to different locations.

As mentioned by de Lagarde et al. and Argudín et al. [16,27], it is possible that mobile genetic elements, such as plasmids, transmit resistance genes from commensal to pathogenic strains that could infect the horse leading to complications during treatments. Although the bacteria founded in the present study are commensals in the horses’ mouth, 89% have high zoonotic potential (e.g., *Klebsiella pneumoniae*, *Escherichia coli*) [28,29,30]. Due to the close contact between humans and horses, it is highly possible that humans will be infected with multidrug-resistant strains.

Sixty-two percent of the commensal microbiota described here, in addition to affecting humans, are commonly found in the environment, namely in the soil, water, and plants (e.g., *Serratia rubidaea*, *Klebsiella pneumoniae*) [28,31]. This means that resistant bacteria and resistance genes can circulate between species and environments, dispersing and accumulating quickly and easily [32]. That fact highlights the correlation between these three pillars (animals, humans, and the environment) that constitute the “*One Health*” concept, proving the excellent framework and important role of the horse. However, the doubt that this situation creates is whether these multi-resistances came from the environment to the horse or whether it was the horse that acquired them by another way and could spread them to the environment. Regardless, the fact that the question remains open means that it is possible to conclude that these bacteria will be in circulation between both (animals and environment), as well as the resistances they carry, with the horse being a vehicle for their dissemination.

Currently, medical care centers are noted as being hotbeds of multidrug-resistant bacteria [33]. Shnaiderman-Torban et al. (2020) [14] compared the percentage of antimicrobial MDR and extended-spectrum beta-lactamase producing Enterobacteriaceae (ESBL-E) shedding rates between horses on farms and hospitals. Hospitalized horses showed a higher percentage of resistance (94.3%) and higher shedding rates than farm horses (89.6%). However, the overall MDR percentage was lower than the one found in our study (96%), which was performed in healthy horses that did not have contact with antimicrobials for a minimum period of 6 months, and some horses who have never had contact with an antibiotic in their lives, making the situation more frightening. The European Medicines Agency (EMA) categorized antibiotics in four classes (A, B, C, and D) according to their impact on public health, and their importance in human medicine [34]. This classification serves as a guide to veterinarians in the choice of antibiotics since many of the practitioners have difficulties in determining whether antimicrobials are necessary for the treatment of a certain patient and which antimicrobial regimen to use [35]. Looking at the percentages of resistance in each class of antibiotics tested on this study, the overall picture does not get any better. β-lactams are one of the most widely used classes of antibiotics in veterinary medicine, belonging to the first-line antibiotics (class D), according to the EMA categorization. Since it has a resistance of 55.4%, this could make therapy significantly less effective, leading clinicians to opt for second (class C) or third-line (class B) antibiotics, which have important roles in human medicine. A little more encouraging is the scenario for tetracyclines and sulfonamides, broad spectrum antibiotics belonging to class D and widely used in horses, which showed lower percentages of resistance, 30.9% and 27.3%, respectively. Aminoglycosides are frequently used, specifically in equine medicine. Given its relevance in this field and the fact that it belongs to class C, 42.7% resistance is of concern. Carbapenems (class A) are a class of antibiotics for almost exclusive use in human medicine in hospital facilities [7,36]; 51.5% of the isolates presented resistance, being more pronounced in meropenem (72.7%) and ertapenem (69%). Shnaiderman-Torban et al. (2020) reports that all the founded isolates were susceptible to imipenem (0% of resistance). The higher resistance to imipenem (12.5%) observed in our study, despite being low, is still significant, since it is an antibiotic rarely used in horses. This can lead us to think of the possible transference of resistant bacteria in a reversed order, from human to horse. Nevertheless, wounds from horse bites or caused during veterinary dentistry procedures are not uncommon and are a way of MDR dissemination, with Litterio et al. (2012) [31] reporting that *Serratia Rubidaea*, one of the MDR bacteria mentioned in our study, was found in an infected wound from a horse bite, which may create complications during treatment. In our study, we found four *Serratia Rubidaea* strains with an alarming antimicrobial resistance profile. Besides being highly resistant to commonly used veterinary antimicrobials, we found that all the isolates tested presented resistance to at least one carbapenem. Meropenem presented with 100% resistance, ertapenem 50% resistance, and only imipenem was free of any kind of resistance.

In a study by Lion et al. (2020) [37], the antimicrobial resistance of equine pathogens between 2016 and 2019 seemed to diminish after implementation of governmental programs to combat antimicrobial resistance in France. In face of such fact, the overall percentage of resistance founded here in Gram-negative commensal microbiota (96%) is indeed worrisome. Comparing one of the most common isolates founded in both studies (*E. coli*), Lion et al. (2020) [37] reported 39.5% resistance to amoxicillin (our study found 60% resistance), 31.4% resistance to amoxicillin conjugated with clavulanic acid (our study found 40% resistance), and the resistance to aminoglycosides was lower than 11.2% (our study found 51.7% resistance). However, *E. coli* isolates founded in our study were more susceptible to sulfonamides (100% susceptibility) and tetracyclines (93.3% susceptibility) compared with 67% and 76%, respectively, susceptibility presented by the mentioned study.

Some of the strategies adopted to minimize MDR were designed to preserve the efficacy of existing antimicrobials, but also to minimize, as much as possible, the spread of multi-resistant agents, with the geographical movement of infected animals being an important driver of the resistance phenomenon [38,39]. The fact that we find such high percentages of multidrug resistance in healthy horses from different regions and those that were sometimes transported to other stables, is an alert sign. Today, the horse plays important roles in society regarding companionship, assisted therapy, and sport [40,41]. Being a sport animal, the horse is often transported across different countries to the competition venues. During transport, there is a high probability of spreading multidrug-resistant bacteria between countries, and even the possibility of inoculating them in countries that are “free” or with lower rates of multidrug resistance strains. As reported by de Lagarde et al. [16], the participation in equestrian events represents a risk factor for shedding MDR isolates. The role of the horse as a companion animal and as an assisted therapy animal should not pass us by, since a study pointed out that owning or being in contact with horses increases the risk for developing some types of MDR bacteria in the people involved [42]. Especially in assisted therapy, where some patients may have some degree of immunosuppression, this fact should be considered in order to adopt adequate biosecurity measures [40].

Antimicrobial misuse and overuse in the human, animal, and environmental sectors as well as the dissemination of resistant bacteria and resistance factors within and between these sectors, are the main causes of antimicrobial resistance [43]. As already mentioned, many studies are related to the evolving antimicrobial resistance in animals that have been admitted to veterinary facilities or that had recent contact with an antibiotic [17,29,30,44]. However, the samples of the present study came from completely healthy horses, located in different parts of the country, and that were free of antimicrobial therapy. These facts decrease the impact of antibiotic selective pressure for MDR development, suggested by several studies [12,45], in these horses and gives relevance to the horse itself, which can be considered as a valuable sentinel that allows us to control the evolution and transmission of multidrug-resistant bacteria between the “One Health triad” since it is in contact with humans, other animals, and the environment, in different geographic locations.

Although there are several studies that have focused on equine microbiota and associated antimicrobial susceptibility, the literature specifically on equine oral commensal microbiota is scarce. The authors underline the importance of this field since oral bacteria are easily cross-transmitted to other species, the environment, and humans, by direct or indirect contact with the horse’s mouth or saliva, which is common during veterinarian and equestrian practices. Furthermore, since bacterial resistance profile changes rapidly [17], continuous surveillance is important. Our data develops the previously confirmed idea that horses should be considered reservoirs of MDR and zoonotic pathogens but also enhances their role as MDR watchers. The transmission of MDR bacteria to all animals and individuals that are in contact with MDR-colonized horses (owners, riders, veterinarians, and others) should not be underestimated [46]. For all these reasons, the authors believe that the data reported on this study is relevant to the “One Health” concept.

For future research, it will be advantageous to perform molecular and genetic studies to evaluate whole genome sequences and resistance genes of these bacteria. Furthermore, it would be beneficial to increase the sample size in order to obtain a larger number of isolates. The small number of isolates hinders the characterization of the commensal oral microbiota of horses in this study.

## 4. Materials and Methods

### 4.1. Animals

During routine oral examinations performed between July and December of 2022 in different regions of Portugal, horses of different breeds and ages, without any kind of systemic or oral disease and deprived of systemic or topical antimicrobial therapy in the last 6 months, were selected for this study. The entire procedure was conducted in accordance with the European Animal Welfare Directives (Directive 98/58/CE).

### 4.2. Sample Collection

Samples were collected as previously described by Gao et al. (2016) [2]. A physical examination was performed to discard any obvious systemic disease. A mild sedation was performed using intravenous detomidine (Domidine^®^, 0.012 mg/Kg, Dechra, Northwich, UK) and butorphanol (Butomidor^®^, 0.025 mg/Kg, Richter Pharma AG, Wels, Austria). An oral speculum was placed and the mouth was washed with a sterile saline (0.9% solution) to remove debris or remaining food. A careful examination of the oral cavity was performed to discard any king of oral disease, such as ulcers on the cheeks or tongue, periodontal disease, diastemata, or caries, since pathological conditions can alter the normal microbiota. Then, using a sterile curette and a swab, a sample was taken from the subgingival space and gingival margin of the tooth 406, without touching the tongue. Samples were placed in tubes with Stuart transport medium, kept at 4 °C, and sent to the Medical Microbiology Laboratory—Antimicrobials, Biocides & Biofilms Unit, Department of Veterinary Sciences, UTAD. Samples were processed in 12–24 h.

### 4.3. Sample Processing

Samples were cultured in tubes with Brain Hearth Infusion (BHI, Oxoid, Hampshire, UK) and incubated at 36 °C for 24 h. After this period, tubes with turbidity were considered positive for bacterial growth and selective and differential growth mediums GSP *Pseudomonas Aeromonas* Selective Agar (Merck, Munich, Germany), Chromocult Coliform Agar (Oxford, Hampshire, UK) and MacConkey (Oxoid, Hampshire, UK), were used for the isolation process.

### 4.4. Species Identification

After subculture of Gram-negative bacteria, the isolates were inoculated into the specific identification cards of the automated VITEK^®^ 2 Compact system (BioMerieux, Paris, France) using the standard protocol: Gram-negative (Vitek^®^ ID-GN card).

### 4.5. Antimicrobial Susceptibility Test

Antibiotic susceptibility phenotypes were determined by the agar diffusion technique based on the Kirby–Bauer method. Bacterial suspensions, with turbidity to 0.5 MacFarland Standard, were spread on Mueller Hinton agar (Oxford, Hampshire, UK) using a sterile cotton swab. After 24 h of incubation at 37 °C, the diameters of antibiotic inhibition of growth were measured, and interpretation of zone diameters were evaluated according to the recommendations of the Clinical and Laboratory Standards Institute (CLSI) (available at https://www.nih.org.pk/wp-content/uploads/2021/02/CLSI-2020.pdf accessed on 12 January 2023) (Table S1) [47]. Twenty-seven antimicrobials of eight different classes that include β-lactams (amoxicillin, amoxicillin/clavulanate, ticarcillin, ticarcillin-clavulanic acid, piperacillin, piperacillin-tazobactam, aztreonam, cephalothin, imipenem, cefoxitin, ceftazidime, cefotaxime, ceftriaxone, cefoperazone, meropenem, and ertapenem), aminoglycosides (kanamycin, tobramycin, gentamicin, and amikacin), quinolones (nalidixic acid and ciprofloxacin), macrolides (erythromycin), sulfonamides (trimethoprim-sulfamethoxazole), tetracyclines (tetracycline), amphenicols (chloramphenicol), and phosphonic acid derivates group (fosfomycin) were tested. Multidrug resistance (MDR) is defined as acquired nonsusceptibility to at least one agent in three or more antimicrobial categories. *Escherichia coli* ATCC 25922 was used as a quality control strain [48].

## 5. Conclusions

This study shows that the equine commensal oral microbiota contains zoonotic and potential pathogenic strains that could be easily widespread through other animals, the environment, and humans, with saliva being a potential vehicle. The overall antimicrobial MDR presented by these bacteria is particularly worrying considering the absent of antimicrobial contact of the horses included, which gives relevance to the transmission of MDR strains and genes between animals. Furthermore, our study confirms the previous concept of the bite as a public health concern since it can inoculate highly resistant and zoonotic strains, which can lead to dangerous and life-threatening infections. Bearing in mind all the mentioned facts, the horse can be considered as a central piece of the One Health concept, with an important role as a sentinel in the surveillance of MDR evolution since it has intimate contact with humans, other animals, and lots of different environments. Furthermore, being frequently transported, this animal can be an important vehicle, having a worldwide impact on multidrug resistance transmission. This study, according to the authors’ knowledge, provides the first report of carbapenems resistance in equine commensal oral microbiota. Since carbapenems are an antimicrobial class particularly used in human medicine hospital settings, this can be problematic when facing infected wounds created by horse bites.

## Figures and Tables

**Table 1:** Resistance to antimicrobial classes tested. Only bacterial species with at least two isolates are presented.

Isolates	n	β-Lactams	Aminoglycosides	Quinolones	Macrolides	Sulfonamides	Tetracyclines	Phenicols	Phosphomycin
*Aeromonas salmonicida*	3	81.3%	66.7%	100%	100%	100%	66.7%	100%	33.3%
*Enterobacter cloacae* *complex*	5	51.3%	80%	30%	80%	20%	20%	20%	0%
*Escherichia coli*	15	38.3%	51.7%	20%	93.3%	6.7%	0%	13.3%	13.3%
*Klebsiella pneumoniae*	3	85.4%	75%	83.3%	100%	66,7%	66.7%	66.7%	66.7%
*Pantoea agglomerans*	5	46.3%	10%	75%	100%	0%	0%	0%	80%
*Pantoea* spp.	2	84.4%	50%	100%	50%	50%	100%	100%	50%
*Pseudomonas fluorescens*	7	67.9%	32.1%	64.3%	85.7%	14.3%	14.3%	28.6%	57.1%
*Pseudomonas putida*	2	78.1%	12.5%	100%	100%	100%	100%	100%	100%
*Serratia rubidaea*	4	62.5%	43.8%	75%	100%	0%	50%	0%	0%

**Table 2:** Resistance to the antimicrobial classes tested. Only bacterial species with one isolate are presented.

Isolates	n	β-Lactams	Aminoglycosides	Quinolones	Macrolides	Sulfonamides	Tetracyclines	Phenicols	Phosphomycin
*Burkholderia gladioli*	1	87.5%	100%	100%	100%	100%	66.7%	100%	0%
*Enterobacter aerogenes*	1	37.5%	0%	50%	0%	0%	0%	0%	0%
*Kluyvera intermedia*	1	50%	0%	0%	0%	0%	0%	0%	100%
*Pasteurella pneumotropica*	1	31.3%	0%	50%	0%	100%	100%	100%	0%
*Pasteurella testadinis*	1	25%	0%	100%	0%	0%	0%	0%	0%
*Pseudomonas paucimobilis*	1	81.3%	0%	100%	100%	100%	100%	100%	100%
*Serratia plymuthica*	1	56.3%	50%	50%	0%	0%	0%	0%	100%
*Shigella sonnei*	1	31.3%%	25%	0%	100%	100%	100%	100%	0%
*Sphingomonas paucimobilis*	1	37.5%	0%	50%	0%	0%	0%	0%	0%

## Data Availability

No new data were created or analyzed in this study. Data sharing is not applicable to this article.

## Supplementary Materials

The following supporting information can be downloaded at: https://www.mdpi.com/article/10.3390/antibiotics12040792/s1, Table S1: Zone diameter breakpoints for the antimicrobials used.
