# Supplementary material for: Equine Gram-Negative Oral Microbiota: An Antimicrobial Resistances Watcher?
Source: Antibiotics (Basel). 2023 Apr 21;12(4):792. doi: 10.3390/antibiotics12040792 (PMC10135200; doi:10.3390/antibiotics12040792)
Supplement: Supplementary file 1 [file antibiotics-12-00792-s001.zip › antibiotics-2316206-supplementary.pdf]

Table S1: Zone diameter breakpoints for the antimicrobials used.

Antimicrobial agent	Zone Diameter Breakpoints (mm)		
	Susceptible	Intermediate	Resistant
AML	≥17	14-16	≤13
AMC	≥18	14-17	≤13
TIC	≥20	15-19	≤14
TIM	≥20	15-19	≤14
PRL	≥21	18-20	≤16
TZP	≥21	18-20	≤17
KF	≥18	14-17	≤13
FOX	≥18	15-17	≤14
CAZ	≥21	18-20	≤17
CTX	≥26	23-25	≤22
CRO	≥23	20-22	≤19
CFP	≥21	16-20	≤15
ATM	≥21	18-20	≤17
IPM	≥23	20-22	≤19
MEM	≥23	20-22	≤19
ETP	≥22	19-21	≤18
NA	≥19	14-18	≤13
CIP	≥26	22-25	≤21
K	≥18	14-17	≤13
TOB	≥15	13-14	≤12
CN	≥15	13-14	≤12
AK	≥17	15-16	≤14
E	≥23	14-22	≤13
SXT	≥16	11-15	≤10
TE	≥15	12-14	≤11
C	≥18	13-17	≤12
FOS	≥16	13-15	≤12
